# Comparison of Population-Based Census versus Birth History for the Estimation of Under-5 Mortality in Niger

**DOI:** 10.4269/ajtmh.22-0725

**Published:** 2023-10-30

**Authors:** Brittany Peterson, Ahmed Mamane Arzika, Abdou Amza, Alio Karamba, Nasser H. Dodo, Nasser Galo, Aboubacar Beidi, Abarchi Moustapha, Elodie Lebas, Catherine Cook, Jeremy D. Keenan, Thomas M. Lietman, Kieran S. O’Brien

**Affiliations:** ^1^ Francis I. Proctor Foundation, University of California, San Francisco, California;; ^2^ Centre de Recherche et Interventions en Santé Publique, Birni N’Gaoure, Niger;; ^3^ Programme Nationale de Santé Oculaire, Niamey, Niger;; ^4^ Department of Ophthalmology, University of California, San Francisco, California;; ^5^ Department of Epidemiology and Biostatistics, University of California, San Francisco, California;; ^6^ Institute for Global Health Sciences, University of California, San Francisco, California

## Abstract

The WHO guidelines on mass distribution of azithromycin for child survival recommend monitoring of mortality to evaluate effectiveness. Trials that contributed evidence to these guidelines used a population-based census to monitor vital status, requiring census workers to visit each household biannually (twice yearly). Birth history is an alternative to the census approach that may be more feasible because it decreases the time and labor needed for mortality monitoring. This study aimed to compare the population-based census (reference standard) and birth history (index test) approaches to estimating mortality among children 1 to 59 months old using data from the Macrolides Oraux pour Réduire les Décès avec un Oeil sur la Résistance (MORDOR) trial. Sixteen communities that received 5 years of biannual census in the MORDOR trial were selected randomly also to receive birth history surveys. The census approach recorded more participants and households than birth history, with correlations more than 0.94 for each. The correlation between number of deaths in each community was 0.84 (95% CI, 0.59–0.94). A comparison of the mortality incidence rate estimated from the census against the under-5 mortality rate estimated from the birth history resulted in a correlation of 0.60 (95% CI, 0.15–0.84). Of the 47% of children who were linked individually to compare vital status from each method, the death status of children had a sensitivity of 80% (95% CI, 73–89) and a specificity of 98% (95% CI, 98–99), comparing birth history to census. Overall birth histories were found to be a reasonable alternative to biannual census for tracking vital status.

## INTRODUCTION

In September 2020, the WHO^1^ released guidelines on mass drug administration (MDA) of azithromycin for child survival. Evidence informing the guidelines includes results from the Macrolides Oraux pour Réduire les Décès avec un Oeil sur la Résistance (MORDOR) cluster-randomized trial in Malawi, Niger, and Tanzania that found a 14% reduction in 1- to 59-month mortality in communities receiving 2 years of biannual azithromycin MDA compared with placebo.^2^ The strongest effects were suggested in subgroups with the highest mortality, including the Niger site and children younger than 2 years old.^2^ Given the risk of increasing antimicrobial resistance,^3^^–^^6^ the guidelines propose this intervention be considered only in high-mortality sub-Saharan African settings and be targeted to children 1 to 11 months old. In addition, the guidelines stipulate that implementation should be accompanied by continuous monitoring of mortality.^1^

To monitor mortality, the MORDOR trial used a population-based census approach in which a study team visited each household in the study area every 6 months to enumerate the target population and track vital status.^2^ Multiple cluster-randomized trials have since been launched in several West African settings to answer additional questions about the effectiveness and implementation of this intervention.^7^^–^^11^ To monitor mortality, each of these trials uses a similar biannual population-based census approach, which enables high coverage and close monitoring of the vital status and person-time at risk in the target population. However, this approach is quite resource intensive because it requires a full-time dedicated study team to travel to the study area every 6 months. As such, biannual census mortality monitoring is unlikely to be feasible as countries transition to programmatic implementation at larger scales. Birth history is a potential alternative to monitoring mortality rates in this setting. As national civil registration and vital statistics systems have limited coverage in many low- and middle-income countries,^12^ child mortality is typically monitored using birth histories included in demographic and health surveys (DHSs) and other large-scale population surveys.^13^^–^^15^ This approach is likely more feasible for program settings, as a single survey is designed to capture births and deaths among children over multiple years.^16^ Limitations of periodic birth history data collection include missing or inaccurate data on dates, ages, and vital status, and the potential for survivor bias.^17^^,^^18^ Numerous studies^19^^–^^22^ have evaluated the validity and reliability of both complete and summary birth or pregnancy histories used in such surveys to estimate mortality via direct and indirect estimation methods, although rarely are high-quality, rigorously collected data on births and deaths available for these comparison. The MORDOR trial presented a unique opportunity to compare estimates of under-5 mortality from birth history surveys to twice-yearly census data collection. We aimed to leverage the MORDOR trial’s biannual census data collection to compare the population-based census and birth history approaches to estimating mortality among children younger than 5 years old.

## MATERIALS AND METHODS

### Study design, setting, and participants.

This diagnostic accuracy study compared estimations of mortality among children younger than 5 years old using two methods: a biannual (twice yearly) population-based census over 5 years (reference standard) and a single, truncated birth history survey covering births over the 10 years prior (index test). Communities that were enrolled in the Niger site of the MORDOR cluster-randomized trial were eligible for inclusion and were selected randomly for participation in the diagnostic accuracy study from the 594 communities included in the MORDOR trial.^2^ Of the three countries included in MORDOR, Niger was selected for inclusion in this study because it was the only site to continue census data collection for a total of 5 years. Eligibility criteria for MORDOR-Niger included location in the Boboye or Loga districts in the Dosso region and a population size of 200 to 2,000 reported on the 2012 national census. The trial included a population-based census every 6 months 1) to enumerate all households, guardians, and children 1 to 59 months of age in the study area; 2) to administer treatment to children 1 to 59 months old; and 3) to monitor mortality in this age group over the 5-year study period. Our study was designed while data collection for the final the census was being completed and before birth history data collection began.

All households in the eligible study area that consented to participate when approached at home by the study team were included in the census. The census included all guardians of children 1 to 59 months old along with the children themselves. Similarly, among the communities selected randomly for participation in this study, all households that consented to participate when approached by the study team were included in the birth history surveys. The birth history surveys included females between 12 and 65 years to capture those who would have been of childbearing age during the 10 years before the survey, as well as to account for uncertainty in age.

### Test methods.

#### Reference standard.

This study considered the biannual population-based census as the reference standard for mortality estimation among children 1 to 59 months old. Every 6 months, census workers visited every household in the trial study area. Local mobilizers were engaged to ensure the census team visited all households. Census data collection included demographic information (name, age, and gender) from the head of household, guardians of children younger than 5 years old, and children younger than 5 years old. In a very rare case during census data collection that the listed caregiver was not the child’s mother, the data collector directed the questions to the mother of the child. The child’s date of birth was recorded using the *carnet de santé* (health card) if available. Otherwise, the child’s age in months was recorded according to the guardian’s report. Follow-up census data collection included a vital status update during which the census worker reported whether the child was alive, had died, had moved, or had an unknown status. If the child had died, date of death was recorded according to the guardian’s report.

Census data collection teams received training before each census, and supervision during data collection. Census data collection teams were unaware of the planned birth history data collection and were not informed of the results while actively collecting data. All census data collection for the MORDOR trial was performed using a custom-designed mobile application (Conexus, Los Gatos, CA) and was uploaded to a Salesforce cloud database (Salesforce, San Francisco, CA).

The community-level incidence rate of mortality among children 1 to 59 months was estimated using the count of deaths that occurred during the 5-year study period among children 1 to 59 months old and person-time at risk for each community. Children were included in the outcome as having died if they were recorded as alive on one census and died on the subsequent census. Person-time at risk was calculated for each inter-census interval and child as the days the child was alive and living in the community between consecutive censuses. Children with a vital status of died, moved, or unknown status contributed to one half of the inter-census period.

As an exploratory comparison, the incidence rate of mortality was used to approximate the under-5 mortality rate (U5MR), with a life table-based conversion using direct observation of person-time contributed by children who died, and assuming an interval of 4 years and 11 months.^23^ We modified the U5MR to remove neonatal mortality because the census data collection only included children 1 to 59 months old. Both the mortality incidence rate and the modified U5MR approximation were used as continuous variables in quantitative comparisons with mortality estimates from birth histories, as described later.

#### Index test.

Communities participating in the MORDOR-Niger trial were selected randomly for birth history surveys. Survey questions were developed based on existing instruments.^14^^,^^16^^,^^17^ Birth history teams comprised of both men and women visited every household in their assigned communities to conduct the survey, working with the same mobilizer team as the census to ensure they visited the same communities as the census team. Those on the birth history team were also members of the census team, so both groups had similar levels of experience collecting these types of data in these communities. If respondents were not available, the team returned to the community at least once to ensure high coverage. Truncated birth histories were conducted for this study to capture births within the 10 years preceding the survey date to include children who would have been 1 to 59 months old and eligible for the MORDOR census during the 5-year study. For each female included in the study, a list of all live births was obtained, including date of birth, gender, survival status, age (if alive), and age at death (if died). *Carnets de santé* were used for confirmation when available. The birth history team was instructed to use the health card in the same way as the census. Thus, if available, the health card was used to report date of birth and age; if not available, the mother was asked to report.

Birth history data collection teams received training before and supervision during data collection. Birth history teams conducted data collection independently of the census and were not provided with information on households, guardians, children, or deaths that were collected previously during the census. Birth history data collection was performed using a CommCare mobile application (Dimagi, Cambridge, MA).

The direct estimation method with a synthetic cohort approach was used to estimate the U5MR, or the probability of dying by age 5 years for each community.^16^^,^^17^^,^^24^ Briefly, probabilities of dying were constructed for specific age intervals: 0, 1 to 2, 3 to 5, 6 to 11, 12 to 23, 24 to 35, 36 to 47, and 48 to 59 months. No adjustment was made for potential heaping of deaths at age 12 months, assuming heaping is equally likely to come from either side of the 12-month threshold. For children with missing information on month of birth, we used a hot-deck imputation approach to assign month to preserve the variation in responses existing in the data and to mimic the approach used in the DHS program.^16^ Day-of-birth imputation was not necessary because this approach only used year and month of birth. All children reported as died had complete information on age at death, and all children had at least year of birth reported, so imputation was only done for month of birth. Component death probabilities were calculated for each age segment and age interval, and accounted for partial exposure to mortality risk. The time period for mortality risk was the 5 years during which MORDOR trials I through III were conducted. Component survival probabilities were then calculated by subtracting each component death probability from one. The product of the component survival probabilities was calculated, subtracted from one, and multiplied by 1,000 to obtain the modified U5MR expressed per 1,000 live births. As with the reference standard, the modified U5MR was used as a continuous variable for quantitative comparisons. Primary analyses excluded the 0-month age group to align with the 1- to 59-month age group targeted in the census. Mortality estimation was conducted using a modified version of the DHS.rates R package (R Foundation for Statistical Computing, Vienna, Austria).^25^ A sensitivity analysis used a fully random approach to impute month of birth.

### Sample size and analysis.

Sample size calculations were conducted to estimate the number of communities required to compare mortality estimates from the two methods with a Pearson’s correlation coefficient.^26^ We anticipated that inclusion of 16 communities would provide 80% power to detect a correlation coefficient of 0.65, assuming a two-sided alpha of 0.05. Using *t *tests, we compared the number of children per community, number of households per community, and distance to the nearest health center between included and excluded communities. We also used negative binomial regression with community count of deaths as the outcome, and person-time as an offset to compare the mortality incidence rate among these communities. Note that to keep the data collection process for each method independent, the birth history teams were not provided with information to allow them to link households or individuals directly to the census data collection. Thus, the sample size calculation and analysis for this study focus on community-level comparisons, rather than individual-level comparisons.

The primary analysis used Pearson’s correlation coefficient to compare the incidence rate of mortality for children 1 to 59 months old estimated by the 5-year census with the modified U5MR estimated by birth histories, excluding the 0-month age group. Scatterplots were used to visualize the estimates from both methods. A subgroup analysis used the same approach to compare mortality estimates among subgroups of communities defined by population size, with small communities defined as having populations less than 500 people and large communities as having populations of 500 people or more. Similarly, we compared the number of households, guardians, children, and deaths captured by each method using scatterplots and Pearson’s correlation coefficient. As an exploratory analysis, we compared visually the modified U5MR approximation from the census to the modified U5MR estimated from the birth histories with Bland-Altman plots and with a two-way mixed-effects model for consistency of measurements to estimate the intraclass correlation coefficient (ICC).^27^ In an additional exploratory analysis, we estimated sensitivity, specificity, and predictive values for vital status (alive or died) reported in birth histories compared with the reference standard. These estimates were conducted at the child level among the subset of children that was able to be linked across methods. A series of steps involving deterministic and probabilistic record linkage were performed using child name, child age, guardian name, and head-of-household name along with the fuzzyjoin package in R (R Foundation for Statistical Computing). First, children were matched based on identical guardian and head-of-household name, with a maximum distance of child name set to five. Then, all remaining head-of-household and guardian pairs were linked, regardless of child name, and child name and age were reviewed manually to link each child in these paired households. Mortality estimation and analyses were conducted in R (R Foundation for Statistical Computing).

## RESULTS

**Figure 1.**
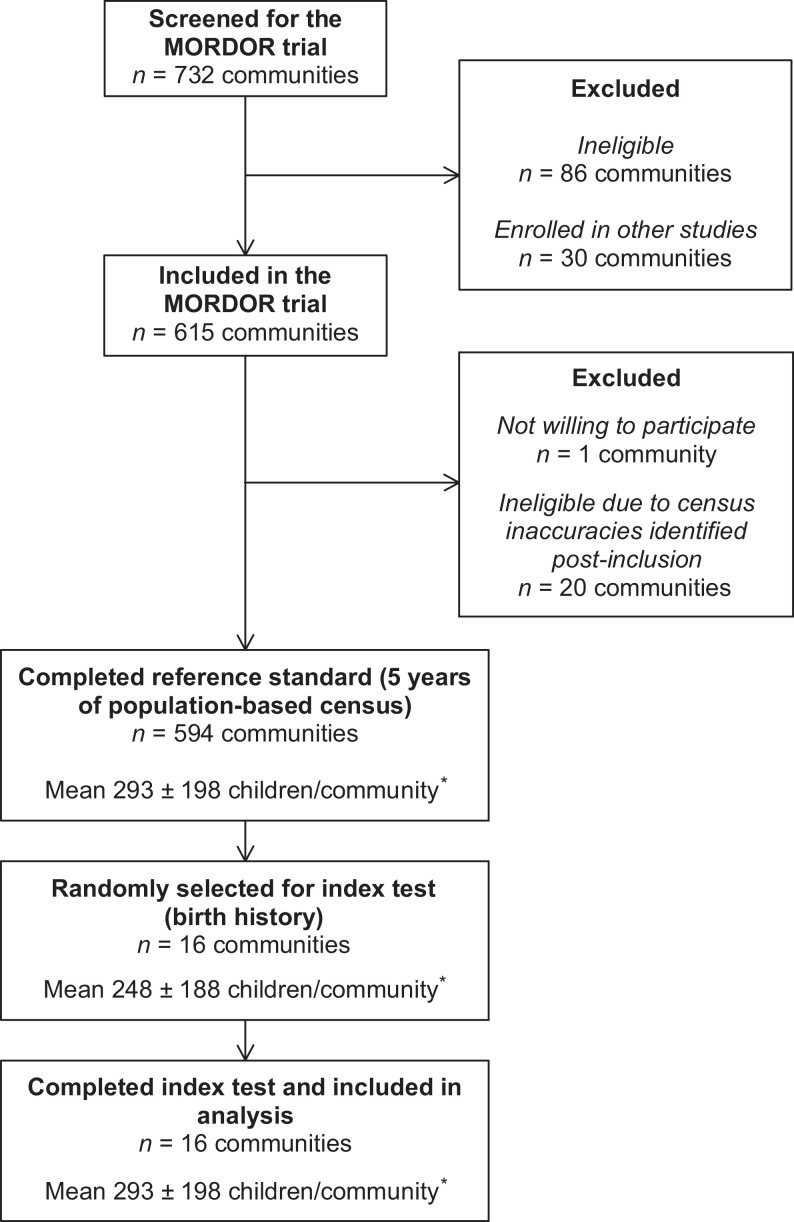
Flow diagram of participants. *Summaries of children and households per community are shown for the total included over the 5-year study. MORDOR = Macrolides Oraux pour Réduire les Décès avec un Oeil sur la Résistance.

The census was conducted every 6 months from December 2014 to June 2020 in 594 communities in Niger. In June 2020, 16 communities were selected randomly to receive birth history surveys, which were conducted from July to October 2020. Figure 1 summarizes the flow of communities from screening for inclusion in the original MORDOR trial through participation in our diagnostic accuracy study. During the 5-year period, the overall group of communities in the trial included an average of 293 ± 198 (SD) children 1 to 59 months old per community. The subset of communities selected for this study included an average of 248 ± 188 children 1 to 59 months old per community over 5 years. The communities included in this study had similar numbers of children, households, and distance to the nearest health center to those not included (*P* = 0.35, 0.19, and 0.84, respectively) (Supplemental Table S1). The incidence of mortality was higher in included compared with excluded groups (*P* = 0.004) (Supplemental Table S1). On average, birth history surveys were conducted 113 ± 47 days after the final census. Respondent populations were similar in the birth history and census data collection, as only female respondents were involved in the birth history data collection and 99.7% of guardians in the census data collection were female.

**Figure 2.**
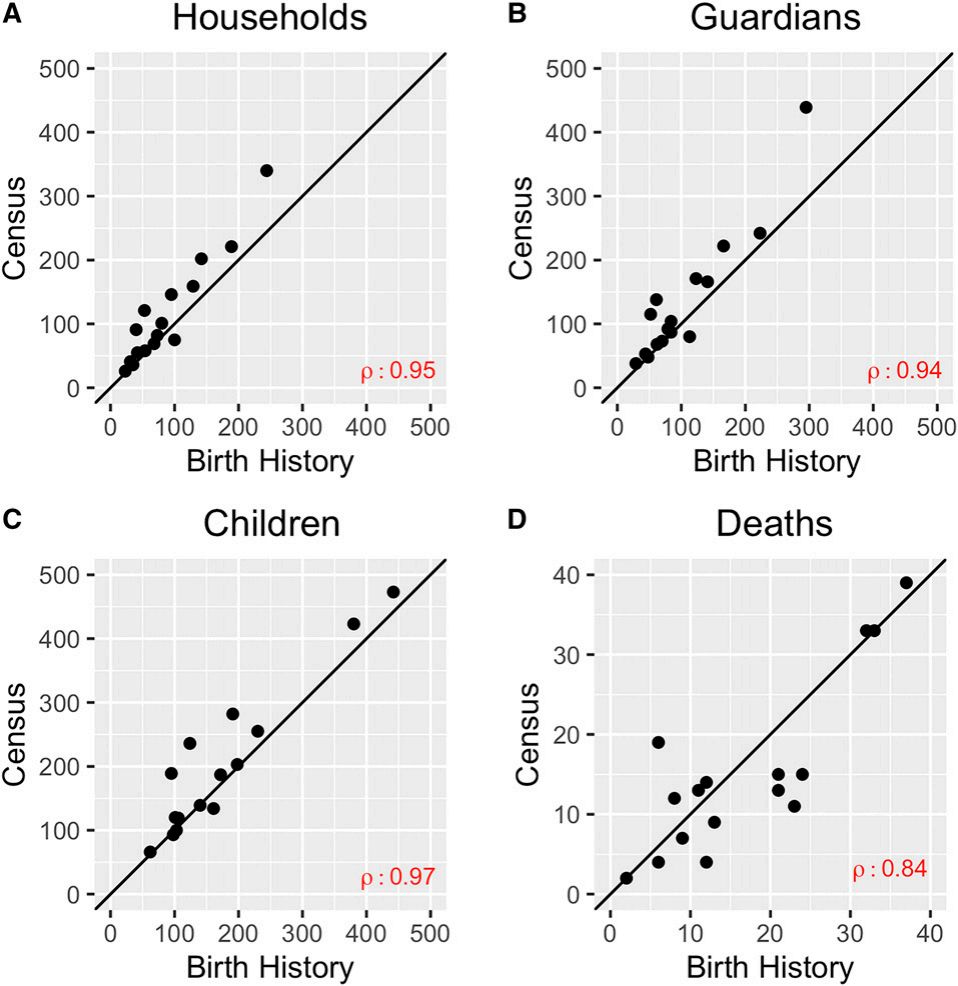
Comparisons of numbers of households (**A**), guardians (**B**), children (**C**), and deaths (**D**) captured by population-based census and birth history approaches (*N* = 16 communities). Pearson’s correlation coefficient shown for each comparison.

Table 1 summarizes the data collected by each method and Figure 2 shows community-level comparisons of the numbers of households, guardians, children, and deaths captured by each approach. Overall, the census and birth history approaches included similar numbers of households, guardians, and children, with the census capturing more households and individuals (Table 1). The correlation was ≥ 0.94 for each of these comparisons (Figure 2). The total number of deaths over the 5-year period captured by the census and birth history was similar, with a correlation coefficient of 0.84 (95% confidence interval [CI], 0.59–0.94) (Table 1). The birth history method resulted in 11 children (0.3%) with unknown vital status who were excluded from the results and 2,186 of 3,211 children (61%) requiring imputation of month of birth. In the census data collection, 10.9% of children had unknown vital status at any time during the 5-year census period. For these 16 communities, five team members collected birth history data, with an average of 1.56 days spent in each community. For the final census data collection in MORDOR, the average number of days spent in a community was 2.19 days, with 32 census workers working in these communities.

**Table 1:** Summary of community-level data collected by the census and birth history methods over a 5-year period (*N* = 16 communities)

Variable	Census			Birth History		
	Overall	Median*	IQR*	Overall	Median*	IQR*
Households	1,684	85	46–147	1,398	71	42–107
Guardians	1,972	98	60–172	1,673	82	59–128
Children	3,640	169	106–276	3,211	151	103–206
Vital status						
Alive	3,373	156	93–254	2,930	128	90–189
Died	243	13	9–16	270	13	9–23

IQR = interquartile range.

* Median and IQR of community.

**Figure 3.**
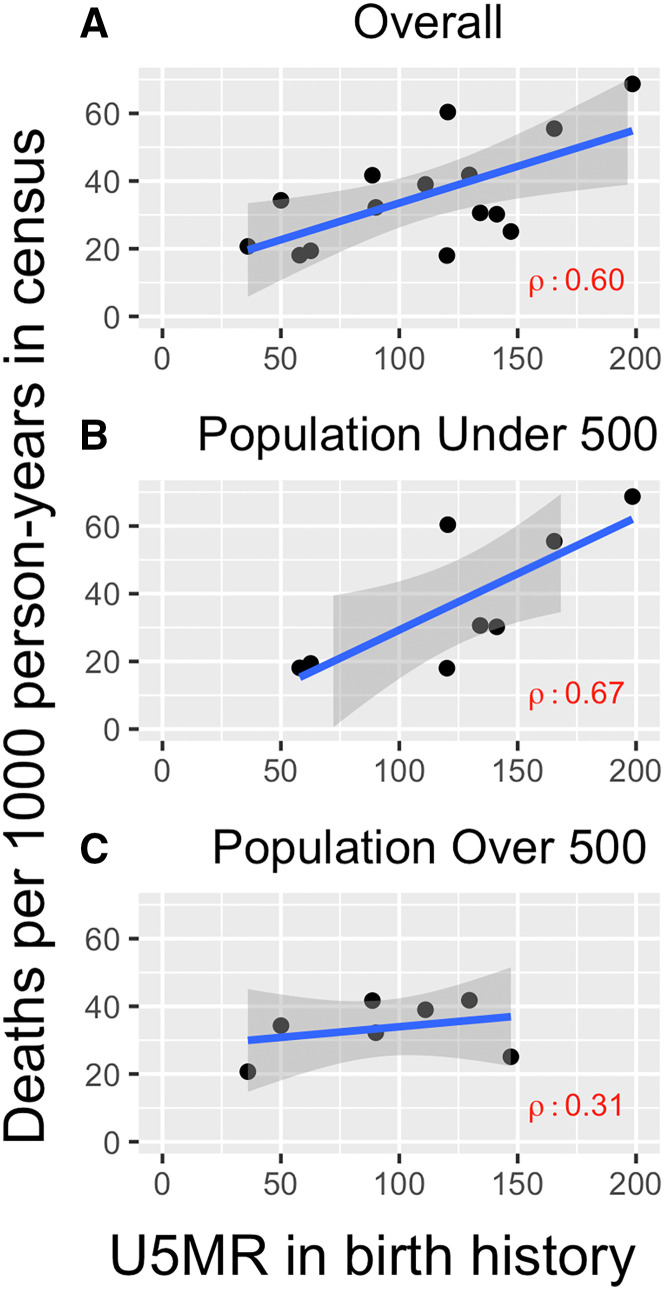
Comparison of mortality estimates for children 1 to 59 months old from population-based census and birth history approaches overall (**A**) and by population size (**B, C**). U5MR = under-5 mortality rate.

**Figure 4.**
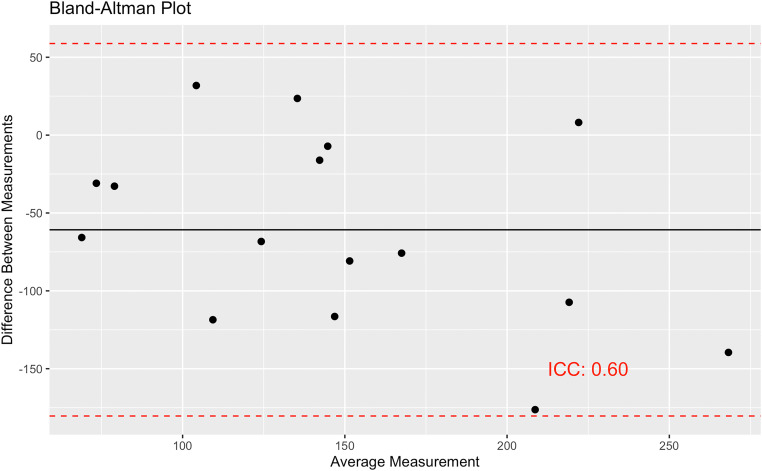
Bland-Alman plots comparing estimates of under-5 mortality from the census and birth history approaches. To estimate under-5 mortality, an approximation was used for data from the census; the synthetic cohort method was used for the birth history data. For both, neonatal mortality was excluded because it was not captured in the census method. ICC = intraclass correlation coefficient.

The community-level comparison of the mortality incidence rate and the modified U5MR estimated using the two approaches resulted in a correlation of 0.60 overall (95% CI, 0.15–0.84) (Figure 3). When examined by subgroup, the correlation was stronger among smaller communities and weaker among larger communities (0.67 versus 0.31, respectively) (Figure 3). As an exploratory analysis, we compared an approximation of the modified U5MR from the census data with the modified U5MR from the birth history data, excluding children younger than 1 month (Figure 4). The Bland-Altman plot indicates that the census approach resulted in an overall higher modified U5MR compared with the birth history approach, but they are moderately consistent, with an ICC of 0.60 (95% CI, 0.17–0.84). Results were similar in sensitivity analyses using a fully random approach to impute month of birth (Supplemental Figures S1 and S2).

At the child level, 1,502 of the total 3,211 children (47%) were linked across the two methods. Sensitivity and specificity for reporting of death in birth history surveys compared with the census were 80% (95% CI, 73–89) and 99% (95% CI, 98–99), respectively. Positive and negative predictive values were 81% (95% CI, 78–82) and 99% (95% CI, 96–100), respectively. Overall accuracy was 98% (95% CI, 97–98).

## DISCUSSION

Child mortality monitoring is an essential component of child survival programs worldwide. Because high-coverage vital registration systems are not yet in place in many low- and middle-income countries, birth histories are included in large population-based surveys such as DHSs to update estimates of child mortality periodically. The availability of alternative sources of vital status data for comparison and validation is rare in such settings. The MORDOR trial’s use of a biannual population-based census over 5 years presents a unique opportunity to evaluate child mortality estimates from birth history against more frequently collected vital status data from a regular census in a rural setting in Niger. We found that overall estimates were reasonably correlated between the two approaches, although birth history captured fewer households and individuals, and also produced lower modified U5MRs, with differences more pronounced in larger communities.

The interpretation of this comparison warrants careful consideration of several key differences in these methods. The reliability of mortality estimates from both methods depends on the full capture of children who have died, the absence of differential birth transference by vital status, and accurate reporting on age of death and number of live births.^16^ Furthermore, some included communities were quite small and may not have stable U5MR estimates. The census involved the consistent presence of the study team with data collection every 6 months throughout the 5-year period. Birth histories were conducted at a single time point, with questions referring to the prior 10 years to include children who would have been included in the census. The census was thus able to track much more closely the individual-level vital status updates over time, resulting in no missing data on birth date or age of death, and likely more accurate birth and death dates than birth history. The difference in approaches also means the census was less likely to be limited by survivor bias, when only surviving mothers are available for reporting.^24^

In addition, the census was able to capture more households than the birth history. Contributing reasons for this may include that the census included treatment distribution to children 1 to 59 months old at the same time as data collection, which may have ensured a greater proportion of the eligible population of guardians and children were present and willing to participate. The consistent presence of the census team may also have encouraged more households to participate in the census data collection, whereas the birth history data collection was not routine. The birth history method also did not capture migration actively; thus, if a household moved out of the community and was not present when the birth history team visited, it was not included. Although it is possible that the census approach overestimated deaths and produced false positives, a previously reported validation of the census approach using verbal autopsy resulted in < 1% false positives (33 false positives in 3,339 total deaths).^28^ Birth history may thus underestimate the population size and mortality compared with the census, based on who is present at the time of data collection and the ability or willingness of guardians to recall births and deaths of children over long periods of time. In fact, birth histories have been shown to be prone to bias in smaller samples, with a tendency to underestimate mortality in high-mortality settings.^16^^,^^29^ To reduce this effect, longer time periods are often used for birth histories,^29^ although we did not have a longer time period from the census for comparison.

Strengths of this study include the 5-year period, which allowed the inclusion of more events compared with shorter time periods. Given the relative rarity of mortality even in high-mortality settings, such analyses are challenging to power adequately unless working in large populations or over long time periods. In addition, this study was nested within a cluster-randomized trial, which ensured that standardized data collection procedures were used by frequently trained study teams for the census, with a similar approach taken for birth histories. The random selection of communities for birth history data collection among the larger pool of communities contributing to the MORDOR trial enables the results to be generalized to similar West African settings. This would also allow smooth scale-up to a programmatic implementation working with larger geographic units. Furthermore, respondent populations were similar between the census and birth history methods.

Limitations of this study include the community-level design. We intentionally did not provide birth history data collectors with a list of households, guardians, or children from the census to allow for an independent comparison of collected data. Because of this approach, no link was created between individual guardians or their children across the methods, and so individual-level indicators of diagnostic accuracy such as sensitivity, specificity, and predictive values could not be calculated for the entire population. Although we did estimate these indicators as an exploratory analysis and saw moderate sensitivity and high specificity, < 50% of children were able to be linked confidently across methods. In addition, it is possible that slightly different populations were captured by the two methods, contributing to the differences seen here. Numerous factors may have contributed to this, including the gap in time between the final census and birth history data collection, seasonal migration, the lack of incentive for participation in birth history compared with the offering of treatment in the census, and the increased hesitancy of populations to participate in the birth history data collection during the early phase of the COVID-19 pandemic (July–October 2020). We also found that the communities selected randomly for birth history had a higher mortality incidence rate than those not included, by chance. We do not anticipate this difference to have a major impact on findings, as a greater number of deaths likely facilitates comparisons given the small geographic units of interest, and all other characteristics were similar.

Population-based census has been the gold-standard approach to estimating mortality in cluster-randomized trials of mass azithromycin distribution for child survival in rural and periurban settings in West Africa. However, this resource-intensive approach is likely not feasible for monitoring effectiveness of programs at scale. We found that birth histories are a reasonable alternative approach to monitoring effectiveness of mass azithromycin distribution in this setting, although trial comparisons may be at risk of lower power to detect effects on mortality, given the potential for lower estimates of the modified U5MR.

## Financial Disclosure

This study was supported by the Bill & Melinda Gates Foundation (OP1032340 and INV-002454) and the Peierls Foundation. The funders had no role in the study design, analysis, interpretation of data, or decision to publish.

## Supplemental files

10.4269/ajtmh.22-0725 Supplemental Materials
