# Supplementary material for: Comparison of Population-Based Census versus Birth History for the Estimation of Under-5 Mortality in Niger
Source: Am J Trop Med Hyg. 2023 Oct 30;109(6):1380–7. doi: 10.4269/ajtmh.22-0725 (PMC10793060; doi:10.4269/ajtmh.22-0725)
Supplement: Supplemental Materials [file tpmd220725.SD1.pdf]

Figure 1. Flow diagram of study communities.

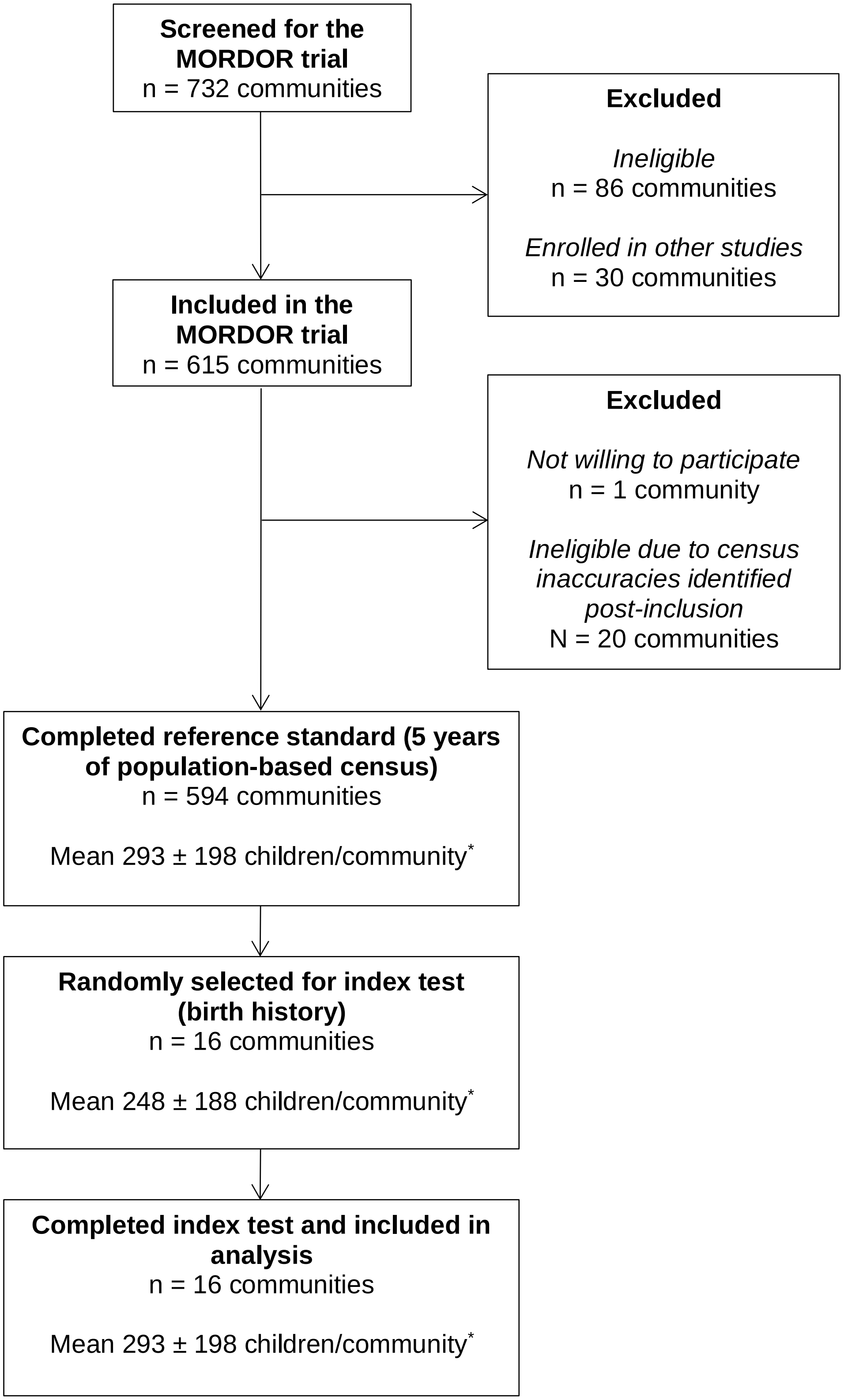

[*]Summaries of children and households per community are shown for the total included over the 5 years study.
